# Caspase 3 involves in neuroplasticity, microglial activation and neurogenesis in the mice hippocampus after intracerebral injection of kainic acid

**DOI:** 10.1186/1423-0127-20-90

**Published:** 2013-12-06

**Authors:** Tsai-Teng Tzeng, Huey-Jen Tsay, Luping Chang, Chia-Lin Hsu, Tzu-Hsuan Lai, Fong-Lee Huang, Young-Ji Shiao

**Affiliations:** 1 Institute of Biopharmaceutical Science, National Yang-Ming University, Taipei 112, Taiwan; 2 Institute of Neuroscience, Brain Research Center, school of life science, National Yang-Ming University, Taipei 112, Taiwan; 3 Division of Basic Chinease Medicine, National Research Institute of Chinese Medicine, Taipei 112, Taiwan; 4 Institute of Anatomy and Cell Biology, National Yang-Ming University, Taipei 112, Taiwan; 5 Ph. D Program for the Clinical Drug Discovery from Botanical Herbs, College of Pharmacy, Taipei Medical University, Taipei 110, Taiwan; 6 National Research Institute of Chinese Medicine, NO. 155-1. Sec. 2, LiNung St., Peitou, Taipei, Taiwan

**Keywords:** Epileptogenesis, Kainic acid, Neurodegeneration, Caspase 3, Gliosis, Neurogenesis, Hippocampus

## Abstract

**Background:**

The roles of caspase 3 on the kainic acid-mediated neurodegeneration, dendritic plasticity alteration, neurogenesis, microglial activation and gliosis are not fully understood. Here, we investigate hippocampal changes using a mouse model that receive a single kainic acid-intracerebral ventricle injection. The effects of caspase 3 inhibition on these changes were detected during a period of 1 to 7 days post kainic acid injection.

**Result:**

Neurodegeneration was assessed by Fluoro-Jade B staining and neuronal nuclei protein (NeuN) immunostaining. Neurogenesis, gliosis, neuritic plasticity alteration and caspase 3 activation were examined using immunohistochemistry. Dendritic plasticity, cleavvage-dependent activation of calcineurin A and glial fibrillary acidic protein cleavage were analyzed by immunoblotting. We found that kainic acid not only induced neurodegeneration but also arouse several caspase 3-mediated molecular and cellular changes including dendritic plasticity, neurogenesis, and gliosis. The acute caspase 3 activation occurred in pyramidal neurons as well as in hilar interneurons. The delayed caspase 3 activation occurred in astrocytes. The co-injection of caspase 3 inhibitor did not rescue kainic acid-mediated neurodegeneration but seriously and reversibly disturb the structural integrity of axon and dendrite. The kainic acid-induced events include microglia activation, the proliferation of radial glial cells, neurogenesis, and calcineurin A cleavage were significantly inhibited by the co-injection of caspase 3 inhibitor, suggesting the direct involvement of caspase 3 in these events. Alternatively, the kainic acid-mediated astrogliosis is not caspase 3-dependent, although caspase 3 cleavage of glial fibrillary acidic protein occurred.

**Conclusions:**

Our results provide the first direct evidence of a causal role of caspase 3 activation in the cellular changes during kainic acid-mediated excitotoxicity. These findings may highlight novel pharmacological strategies to arrest disease progression and control seizures that are refractory to classical anticonvulsant treatment.

## Background

Epileptogenesis is the process of epilepsy development which is characterized by recurrent seizures following an initial insult, such as status epilepticus (SE). This process requires intricate molecular, cellular and hippocampal network reorganization before the first spontaneous seizure occurs. The changes among epileptogenesis include neurodegeneration, neurogenesis, axonal sprouting, dendritic plasticity alteration, and gliosis [1-6].

Kindling is a commonly used model for the development of seizures and epilepsy. kainic acid (KA) is one of the most common chemoconvulsants used to create SE models of temporal lobe epilepsy (TLE). Hippocampal lesions in this model are similar to the hippocampal sclerosis observed in humans with TLE [7-10]. KA is commonly administered systemically to cause sustained neuronal depolarization and seizure generation with a high mortality rate [11]. To reduce mortality, KA may be alternatively injected into lateral ventricle [12].

Hippocampus is an important structure in the pathophysiology of epilepsy. Principal neurons and interneurons are two major groups of neurons in the hippocampal cortex. Most of the principal neurons, such as pyramidal neurons, form excitatory synapse on the remote neurons, whereas the interneurons form inhibitory synapses on principal neurons and other interneurons to prevent the generation of convulsions. Histologically, hippocampal cortex can be divided into CA1-CA4 fields, which contains small pyramidal neurons. The circulation of nerve impulses is formed between CA1-CA4 and enthorinal cortex [13]. The initial limbic seizures increase hippocampal neurogenesis from radial glial cells [14,15]. Prolonged seizures, however, result in aberrant migration and connection of newly born neurons [16,17] and lead to recurrent excitatory circuitry [18]. Conversely, chronic recurrent spontaneous seizures are associated with substantially reduced neurogenesis that coexists with learning and memory impairments [19].

The involvement of astrogliosis in epileptogenesis may be attributable to altered dynamic signaling between neurons, astrocytes and several astrocytic membrane proteins [20]. SE may stimulate reactive astrocytes to proliferate and express more glial fibrillary acidic protein (GFAP) [21], which is associated with altered glutamate uptake and calcium signaling [22]. Morphologically, SE causes thickening and overlapping of astroglial processes and loss of astroglial domains [23]. Nevertheless, the molecular link between initial insults and later changes including neurodegeneration, neurogenesis, synaptic plasticity alteration, and astrogliosis, remains to be elucidated.

Caspase 3 is implicated in the regulation of synaptic plasticity alteration [24], cytoskeletal remodeling [25], and the differentiation of glial cells [26] and stem cells [27]. Notably, localized caspase 3 activity that causes synaptic failure has been observed *in vitro*[28], but the molecular mechanism linking caspase 3 activity to synaptic loss in epileptogenesis is unclear. Furthermore, although caspase 3-mediated cleavage of astrocytic GFAP has been previously detected in reactive or degenerating astrocytes [27,29], the effects of caspase 3 on reactive astrocytes or radial glial cells during epileptogenesis require further investigation. To verify the role of caspase 3 in neurodegeneration, neurogenesis, synaptic plasticity, and astrogliosis during the early phase of epileptogenesis, the specific inhibitor of caspase 3 was applied onto an SE-induced epilepsy model which kainic acid (KA) is administered via intracerebral ventricle (icv) injection. Our data suggests that caspase 3 activity is crucial for cellular alterations during epileptogenesis.

## Methods

### Animals and treatment

The Institutional Animal Care and Use Committee at the National Research Institution of Chinese Medicine approved the animal protocol (IACUC No: P-99-18). The outbred CD-1 (ICR) mice are selected for this study due to that they are vulnerable to neurodegeneration [30]. Six week-old male CD-1 mice were housed for 1 week under standard conditions at 25 ± 2°C with a 12-h light/dark cycle and were allowed free access to water and standard chow. Administration (icv) of KA (Merck) was performed unilaterally on male CD-1 mice (6 weeks old). The mice were anesthetized with intraperitoneal (ip) chloral hydrate (Sigma; 0.4 g/kg body weight, maintained with 0.1 g/kg hourly) and fixed into a stereotaxic apparatus. The dorsal surface of the skull was exposed with a midline incision, and a burr hole was drilled at the following coordinates: anteroposterior, 0.22 mm caudal to bregma and 1 mm right lateral to midline. A 10-μl Hamilton syringe fitted with a 25-gauge needle and filled with KA alone or combined with caspase 3 inhibitor (DEVD-CHO, Insolution Caspase-3 inhibitor I, cell permeable, 1 mg/100 μl, Merck) solution in saline was placed over the burr hole and lowered 2.5 mm into the surface of the brain, and the solution was injected at a rate of 0.2 μl/min. The needle was then left in place for 2 min before it was slowly retracted. Control animals were injected with saline. The first seizure lasting for at least half hour was used to identify the occurrence of SE. The proper KA amount for KA-icv-injection experiment was determined by the profile of KA-induced seizure after the mice to regain consciousness from anesthetization. The seizure profile was determined according to Racine’s scale [31]: stage 0, no response or behavior arrest; stage 1, chewing or facial twitches; stage 2, chewing and head nodding or wet dog shakes; stage 3, unilateral forelimb clonus; stage 4, bilateral forelimb clonus and rearing; stage 5, bilateral forelimb clonus, rearing and falling. The proper KA amount was then determined as 2 μl (0.4 μg). The proper caspase 3 inhibitor amount was set as 1 μl (10 μg) base on the previous study [32,33].

### Tissue processing

At day 1, 3, 5, and 7 post KA-injection and day 7 post vehicle-injection as control, mice were deeply anesthetized with chloral hydrate and perfused through the heart with 30 ml of saline followed by 30 ml of fixative solution containing 4% formaldehyde in saline. The brain was removed, post-fixed in 4% formaldehyde for 18 h at 4°C and cryoprotected in 30% sucrose solution in 0.1 M phosphate-buffered saline (PBS). A cryostat was used to cut 30-μm coronal sections through the dorsal hippocampus, which were collected serially in PBS.

### Fluoro-Jade B staining

Fluoro-Jade B (FJB) has been used for the histological staining of degenerating neurons [34,35]. For staining the KA-mediated neurodegeneration, brain sections were mounted on polylysine-coated glass slides and fully dried. The sections were rehydrated by immersion in 100% ethanol, 70% ethanol, and distilled water for 1 min each. The slides were then transferred to a solution of 0.06% potassium permanganate for 15 min on a horizontal shaker, rinsed for 1 min in distilled water, and then incubated in a solution of 0.0004% FJB (Invitrogen) for 20 min at room temperature. The sections were rinsed 3 times for 1 min each in distilled water, and the slides were fully dried. The slides were cleared by immersion in xylene for at least 1 min before cover slipping. Digital images were acquired using a confocal laser scanning microscope (Leica CS SP, Waltlar, Germany and Zeiss LSM780, Jena, Germany) with excitation at 488 nm and emission at 525 nm.

### Immunohistochemistry

Brain sections were incubated in blocking solution (PBS containing 5% normal donkey serum, 2% Triton X-100, 0.02% bovine serum albumin, BSA) overnight at 4°C and left overnight at 4°C in staining solution (PBS containing 5% normal goat serum, 0.25% Triton X-100, 0.02% BSA) with primary antibodies, including mouse monoclonal antibody to GFAP (Invitrogen); rabbit polyclonal antibody to doublecortin (Abcam); goat monoclonal antibody to ionized calcium-binding adaptor molecule-1 (Iba-1, Abcam); and rabbit polyclonal antibody to active caspase 3 (R&D Systems). Sections were then incubated in staining solution containing Hoechst33258 (Invitrogen, 2 μg/ml), Fluorescein isothiocyanate-conjugated donkey anti-mouse IgG and RRX-conjugated donkey anti-rabbit IgG or cy5-conjugated donkey anti-goat IgG (1:200; Jackson ImmunoResearch) in the dark overnight at 4°C. Sections were then washed in PBS and mounted with Aqua Poly/Mount (Polyscience Inc., Warrington, PA, USA).

### Quantitative immunofluorescence analysis

All analyses were calculated within a field (250 × 250 μm^2^) of Cornu Ammonis (CA)1, CA3 and dentate gyrus (DG) of 3 series hippocampal slices every brain in a range of bregma −1.34 to −1.49. The numbers of FJB-positive neurons were manually counted. Because the control sample did not have any degenerating cells, the number of FJB-positive cells at day 1 post KA injection was defined as 100% degeneration, and the values from other time points were calculated as the percentage relative to it. MetaMorph Image System and Microsoft Excel were used to assess the images and quantify the relative florescent intensity. The grayscale values relative to the highest concentration of fluorescence and the lack of fluorescent marker in the section were defined as 100% and 0% density values, respectively. The expression of GFAP and Iba-1 was calculated from the immunoreactivity (IR) of each protein that displayed as the optical density (arbitrary unit) of each image.

### Postsynaptic density (PSD) preparation

The brain was rapidly dissected, and the hippocampus was homogenized in 10 volumes of H-Buffer (320 mM sucrose, 2 mM EDTA, 20 mM Tris–HCl [pH 7.4], 1 mM PMSF, 5 μg/ml leupeptin, 5 μg/ml aprotinin) with 30 strokes with a tight-fitting glass Dounce tissue grinder (5 ml, Wheaton). The homogenate was centrifuged at 1,000 × *g* for 10 min, and the resulting supernatant was centrifuged at 13,000 × *g* for 20 min. The pellet was resuspended in an equal volume of TET buffer (1% Triton-X 100, 2 mM EDTA, 20 mM Tris–HCl [pH 7.4], 1 mM PMSF, 5 μg/ml leupeptin, 5 μg/ml aprotinin) and agitated for 1 hr at 4°C. The extracts were centrifuged at 100,000 × *g* for 1 h. The resulting pellet was resuspended in 0.1 volume of buffer containing 1% SDS, 2 mM EDTA, and 20 mM Tris–HCl (pH 7.4). Then, 0.9 volume of TET buffer was added, and the extracts were agitated for 1 hr at 4°C, sonicated and incubated on ice for 20 min. The samples were centrifuged at 11,500 × *g* for 10 min, and the protein concentration of the resulting supernatant was determined.

### Immunoblots

For Western blot analysis, samples (6 μg protein) were separated by sodium dodecyl sulfate-polyacrylamide gel electrophoresis (15% gels) and were then transferred to PVDF membranes. The primary antibodies used were as follows: mouse monoclonal antibody to GFAP (BD Bioscience), PSD-95 (Millipore), actin (Invitrogen), rabbit polyclonal antibody to glutamate receptor 1 (GluR1), NMDA receptor 1 (NR1), and calcineurin A (CN-A) (Millipore). The secondary antibodies were anti-rabbit IgG antibody conjugated with horseradish peroxidase (HRP; GE Healthcare) and anti-mouse IgG antibody conjugated with HRP (Jackson ImmunoResearch). Enhanced chemiluminescence detection reagents (GE Healthcare) were used for detection. Bands were quantified using Fujifilm LAS-3000 Luminescent Image Analyzer (Tokyo, Japan).

### Statistical analysis

The results are expressed as the mean ± standard deviation (S.D.) and were analyzed by analysis of variance (ANOVA) with post-hoc Bonferroni multiple comparisons tests.

## Results

### The neurodegeneration occurred in the hippocampus of the KA-icv-injected mice

**Figure 1:**
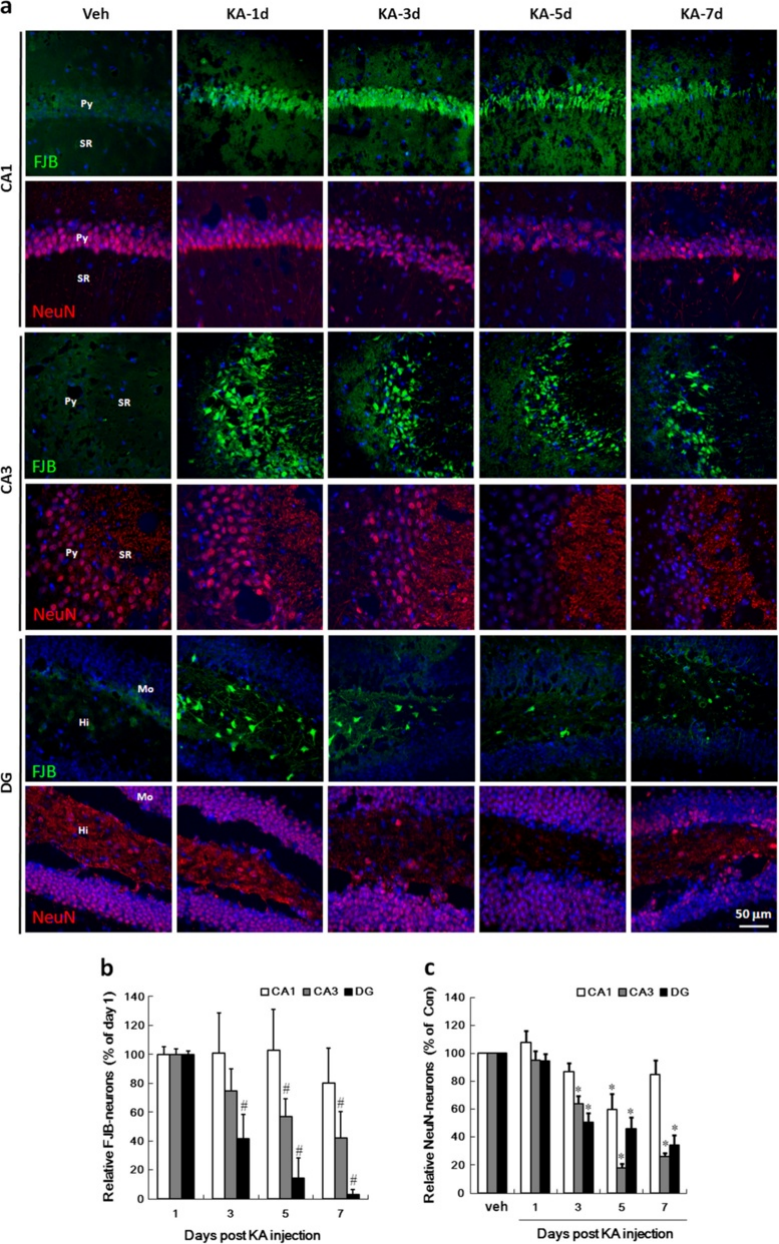
**The neurodegeneration in the hippocampus of the KA-icv-injected mice.** CD-1 mice received KA-icv-injections and were sacrificed at day 1, 3, 5, and 7 post KA-injection (KA-d1, KA-d3, KA-d5, and KA-d7). For control, the mice were sacrificed at day 7 post vehicle-injection (veh). The neurodegeneration in the ipsilateral side of CA1, CA3, and DG was examined by FJB-staining (FJB, green) and immunostaining with anti-NeuN (NeuN, red) antibody. Cell nuclei were stained with Hoechst 33258 (blue). Panel **(a)** shows the representative fluorescent images of the ipsilateral side of CA1, CA3 and DG. The location of pyramidal layer (Py), striatum radiatum (SR). Molecular layer (Mo), and Hilus (Hi) are indicated in the images of the left lane of the panels. The number of FJB-positive **(b)** and NeuN-positive **(c)** neurons in the ipsilateral side of CA1 (white columns), CA3 (grey columns), and DG (black columns) were calculated using MetaMorph software. The results are the mean ± S.D. from 8 images. The data in panel **(b)** are the percentages relative to the day 1 post injection. The data in panel **(c)** are the percentages relative to the vehicle injection. Significant differences between day 1 post injection and day 3, 5 and 7 post injection in panel **(b)** are indicated by #, P < 0.001. Significant differences between vehicle injection and KA injection in panel **(c)** are indicated by *, P < 0.001.

To explore the neurodegenerative process after KA-injection, FJB staining of hippocampus was performed (Figure 1a, b). The results show that the neurons were quickly labeled by FJB (FJB-neurons) at day 1 post KA-injection. The major FJB-neurons include the pyramidal neurons in CA1/3, and the interneurons in DG-hilus. During the period of day 3 to 7 post KA-injection, the number of the FJB-neurons was reduced time-dependently. The decrease rate of the FJB-neurons in DG-hilus was faster than that in CA3. Alternatively, the number of FJB-neurons in CA1 is not significantly altered (Figure 1b). We use NeuN as another marker to verify the neurodegeneration. Our result observe a prominent loss of NeuN in CA3 and DG beginning at day 3 post KA-injection, and a transient but reversible loss at day 5 post KA-injection in CA1 (Figure 1a and 1c).

### Caspase 3 activation occurred in both neurons and glial cells in the hippocampus of KA-icv-injected mice

**Figure 2:**
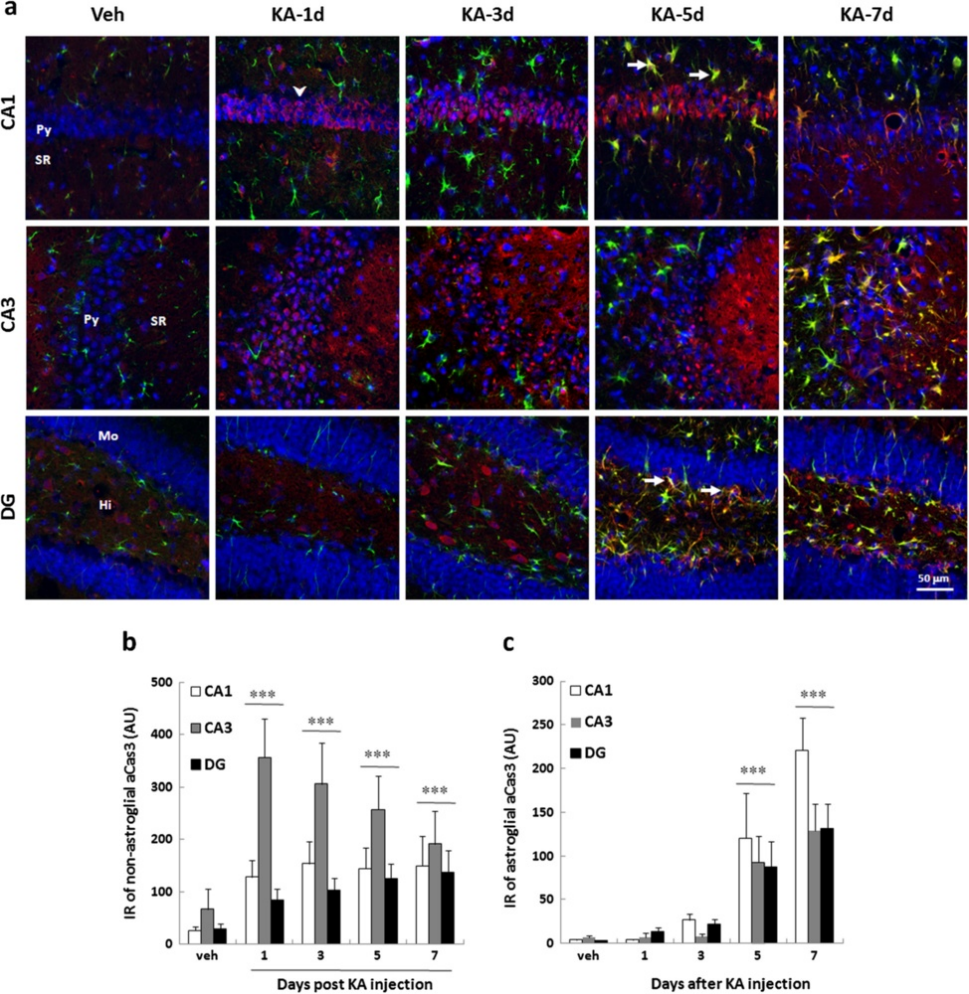
**The caspase 3 activation in the hippocampus of the KA-icv-injected mice.** CD-1 mice received KA-icv-injections and were sacrificed at day 1, 3, 5, and 7 post KA-injection (KA-d1, KA-d3, KA-d5, and KA-d7). For control, the mice were sacrificed at day 7 post vehicle-injection (veh). The caspase 3 activation and the reactive astrocytes in the ipsilateral side of CA1, CA3, and DG was examined by immunostaining with anti-active caspase 3 (aCas3, red) and anti-GFAP (green) antibodies. Cell nuclei were stained with Hoechst 33258 (blue). Panel **(a)** shows the representative photograph of the partial co-localization of active caspase 3 (aCas3) and GFAP in the ipsilateral side of CA1and DG at day 5 and 7 post KA-injection. The location of pyramidal layer (Py), striatum radiatum (SR). Molecular layer (Mo), and Hilus (Hi) are indicated in left lane of the panels. Arrows indicate the representative colocalization of aCas3-IR with astrocytes and radial glial cells in CA1 and DG, respectively. Arrow heads indicate the activation of capsease 3 in pyramidal neurons in CA1. The IR of non-astroglial **(b)**, astroglial **(c)** active caspase 3 in the ipsilateral side of CA1 (white columns), CA3 (grey columns), and DG (black columns) were calculated using MetaMorph software. The data are presented as the percentage relative to the vehicle injection. The results are the mean ± S.D. from 8 images. The data are the percentages relative to the vehicle injection. Significant differences between vehicle injection and KA injection are indicated by ***, P < 0.001.

To verify the contribution of the caspase-dependent apoptosis on KA-mediated neurodegeneration, we performed immunostaining by anti-active caspase 3 antibody (Figure 2). The results show that caspase 3 may be activated in neurons and glial cells successively. During the period of day 1 to 3 post KA-injection, caspase 3 was activated in the cell body of pyramidal neurons in both CA1 and CA3. In the CA3, the colocalization of active caspase 3 with the neurites of pyramidal neurons was observed in the stratum radiatum. In DG, active caspase 3 was found in the hilar interneurons at day 3 post KA-injection (Figure 2a, 2b). During the period of day 5 to 7 post KA-injection, active caspase 3 was observed in astrocytes and the radial glial cells in subgranular zoon (SGZ) of DG (Figure 2a, 2c).

### Astrogliosis and microglial activation is promoted in the hippocampus of KA-icv-injected mice

**Figure 3:**
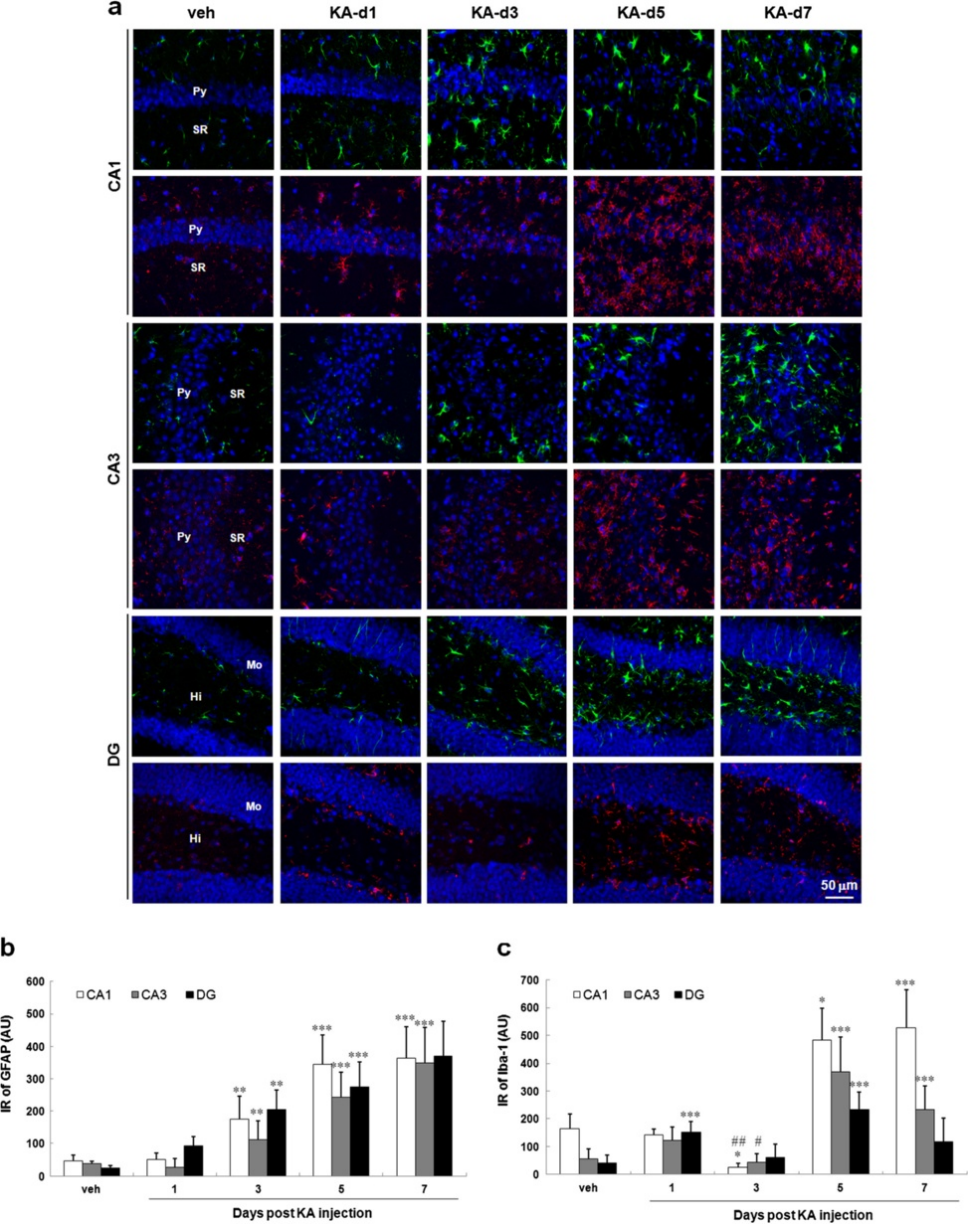
**The microglial activation in the hippocampus of KA-icv-injected mice.** CD-1 mice received KA-icv-injections and were sacrificed at day 1, 3, 5, and 7 post KA-injection (KA-d1, KA-d3, KA-d5, KA-d7). For control, the mice were sacrificed at day 7 post vehicle-injection (veh). The microglial activation in the ipsilateral side of CA1, CA3, and DG was examined by immunostaining with anti-Iba-1 (red) and anti-GFAP (green) antibodies. Cell nuclei were stained with Hoechst 33258 (blue). The representative images of the ipsilateral side of CA1, CA3 and DG area are shown **(a)**. The location of pyramidal layer (Py), striatum radiatum (SR). Molecular layer (Mo), and Hilus (Hi) are indicated in the left lane of the panels. The IR of GFAP **(b)** and Iba-1 **(c)** in 250 μm × 250 μm field in CA1 (white columns), CA3 (gray columns) and DG (black columns) were calculated. The results are the mean ± S.D. from 8 images. Significant differences between the control (veh) and the KA injection are indicated by *, P < 0.05; **, P < 0.01; ***, P < 0.001. Significant differences between day 1 post KA injection and day 3 post KA injection in panel d are indicated by #, P < 0.05; ##, P < 0.01.

To assess hippocampal astrogliosis and microglial activation, we performed immunostaining using anti-GFAP and anti-Iba-1 antibodies (Figure 3a). Astrocytes in the hippocampus of control mice displayed small somata with thin processes morphologies. After KA-injection, the cell bodies of astrocytes were enlarged, and their cell processes were thickened. GFAP immunoreactivity (IR) was significantly increased at day 3 post KA-injection and with the highest expression at day 7 post KA-injection (Figure 3b). The relative IR per field of view of GFAP increased by 7.1-, 13.4- and 14.5-fold of the vehicle-injected mice in CA1, CA3, and DG, respectively. The number of GFAP-stain radial glial cells in SGZ is also increased after KA-injection.

Microglia in the hippocampus of control mice displayed ramified morphologies. After KA injection, the cell bodies of a microglia were enlarged, and their cell processes were thickened. However, the pattern of cell number increase of microglia was different as comparing with astrocytes (Figure 3a). The increase of Iba-1 IR was biphasic after KA-injection (Figure 3c). The first phase occurred at day 1 post KA-injection, and the second phase peaked at day 5 post KA injection. Between these two peaked phases, Iba-1 IR fall in a trough at day 3 post KA-injection. In the second phase, the IR increased by 19.7-, 8.6- and 4.0-fold of that at day 3 post KA-injection in CA1, CA3, and DG, respectively.

### Caspase 3 activation involves in neurite structure alteration but not in neurodegeneration

**Figure 4:**
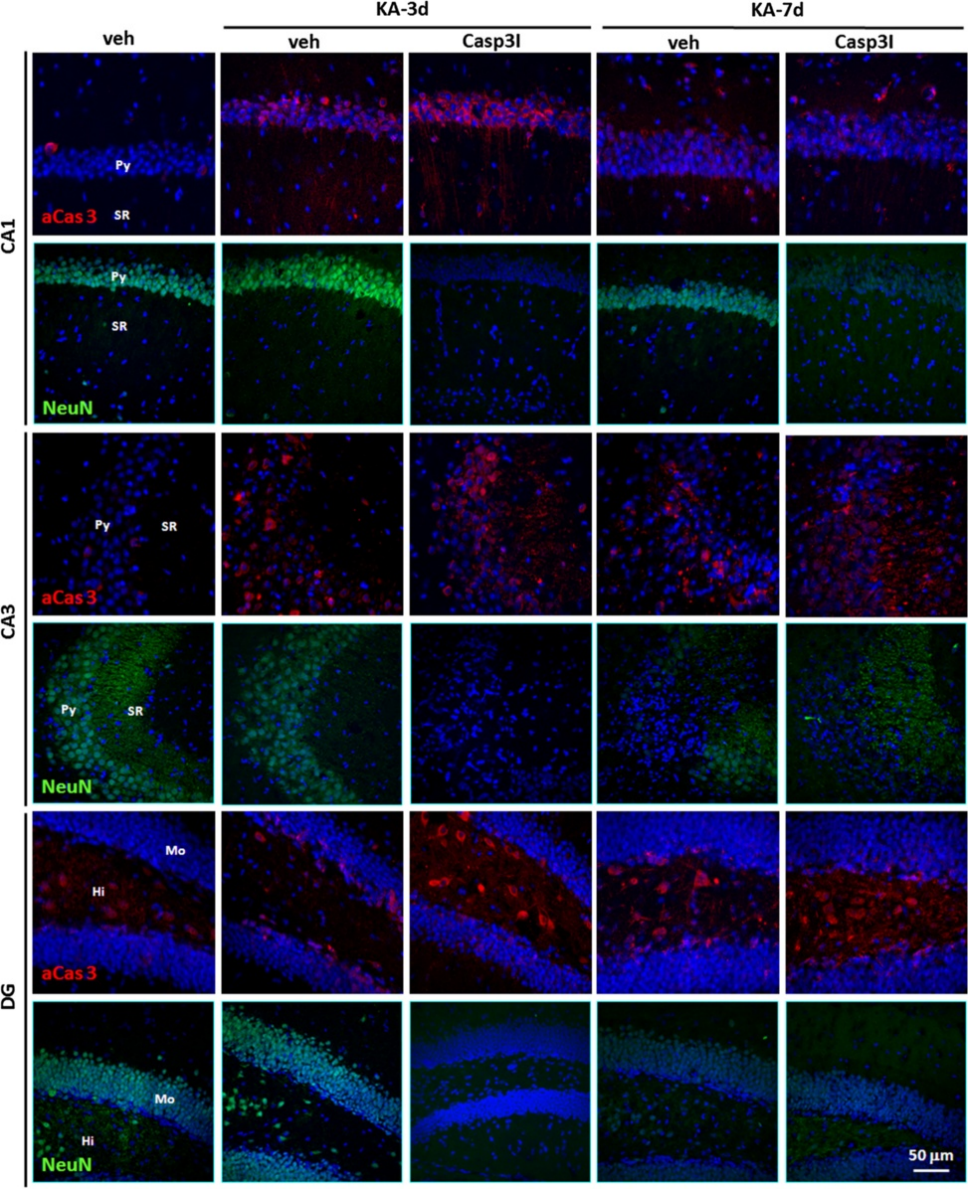
**Caspase 3 inhibitor does not prevent the neurodegeneration in the hippocampus of the KA-icv-injected mice.** CD-1 mice received KA-icv-injections alone or co-injection of KA and caspase 3 inhibitor (Cas3I) or vehicle (veh), and were sacrificed at day 3 and 7 post KA-injection (KA-d3 and KA-d7). For control, the mice were sacrificed at day 7 post vehicle-injection (veh). The neurodegeneration in the ipsilateral side was examined by immunostaining with anti-active caspase 3 (aCas 3, red) and anti-NeuN (NeuN, green) antibodies. Cell nuclei were stained with Hoechst 33258 (blue). The representative fluorescent images of the ipsilateral side of CA1, CA3 and DG are shown. The location of pyramidal layer (Py), striatum radiatum (SR). Molecular layer (Mo), and Hilus (Hi) are indicated in the left lane of the panels.

**Figure 5:**
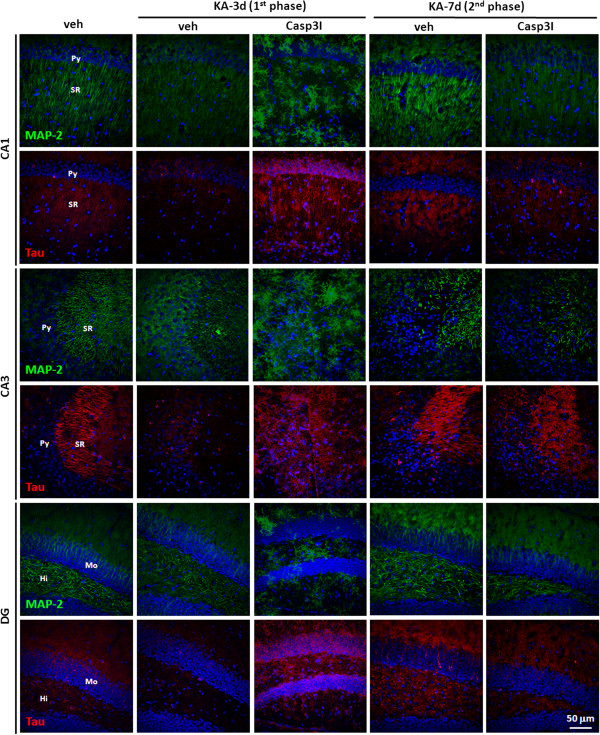
**Caspase 3 inhibitor, but not KA, induces a reversible neuritic alteration in the hippocampus of the KA-icv-injected mice.** CD-1 mice received KA-icv-injections alone or co-injection of KA and caspase 3 inhibitor (Cas3I) or vehicle (veh), and were sacrificed at day 3 and 7 post KA-injection (KA-d3 and KA-d7). For control, the mice were sacrificed at day 7 post vehicle-injection (veh). The neurites in the ipsilateral side of CA1 was examined by immunostaining with anti-MAP-2 (green) and anti tau-protein (Tau, red) antibodies. Cell nuclei were stained with Hoechst 33258 (blue). The representative fluorescent images of the ipsilateral side of CA1 are shown. The location of pyramidal layer (Py), striatum radiatum (SR). Molecular layer (Mo), and Hilus (Hi) are indicated in the left lane of the panels.

The caspase 3 inhibitor (insolution Casapase-3 Inhibitor I, Merck) was then employed to elucidate the role of caspase 3 activation on KA-mediated neurodegeneration. The inhibiting target of caspase 3 inhibitor is the activity of the active caspase 3, but not the activation of caspase 3. Therefore, after co-injection of KA and caspase 3 inhibitor, the biphasic activation of caspase 3 was not altered by caspase 3 inhibitor (Figure 4). NeuN was completely loss at day 3 post KA-injection, which did not occur as KA injection alone. The structural integrity of axon and dendrite was further examined by immunostaining using anti-MAP-2 and anti-tau antibody, respectively [36]. The results indicate that the structural integrity of axon and dendrite is mildly affected after KA-injection. The co-injection of KA and caspase 3 inhibitor, however, seriously disturb the structural integrity of axon and dendrite at day 3 post KA-caspase 3-injection in CA1, CA3 and DG (Figure 5). The disturbance is recovered at day 7 post KA-caspase 3-injection.

### Caspase 3 activation is involved in the GFAP cleavage in the hippocampus of KA-icv-injected mice

**Figure 6:**
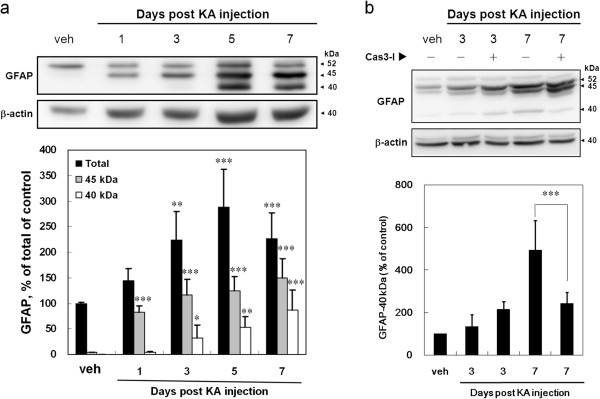
**The cleavage of GFAP in the hippocampus of KA-icv-injected mice is caspase 3 dependent. (a)** CD-1 mice received KA-icv-injections and were sacrificed at day 1, 3, 5, and 7 post KA-injection. For control, the mice were sacrificed at day 7 post vehicle-injection (veh). Hippocampus was removed and homogenized, and the lysates were analyzed by immunoblotting. The top part shows a representative immunoblot of the full length GFAP (52 kD) and its proteolytic fragments (45 and 40 kD). β-actin was used as internal standard. The bottom panel shows the level of total GFAP protein (black columns), 45 kDa-fragment (gray columns) and 40 kDa-fragment (white columns) relative to the level of 52-kD GFAP in vehicle treated mice (veh). The results are the mean ± S.D. from five independent experiments. Significant differences between the control (veh) and the KA injection are indicated by *, P < 0.05; **, P < 0.01; ***, P < 0.001. **(b)** ICR mice received KA-icv-injections alone or co-injection of KA and caspase 3 inhibitor (Cas3I) or vehicle (veh), and were sacrificed at day 3 and 7 post KA-injection. Hippocampal lysates were analyzed by immunoblotting. The top panel shows a representative immunoblot of GFAP and its fragments. β-actin was used as internal standard. The bottom panel shows the level of the 40 kD fragment at day 3 and 7 post KA-injection relative to the level of 40-kD GFAP in the control (veh). The results are the mean ± S.D. from three independent experiments. Significant differences between the mice treated with and without caspase 3 inhibitor are indicated by ***, P < 0.001.

GFAP is the building block of astroglial intermediate filaments. Levels of GFAP and its cleavage have been related to reactive gliosis and astroglial apoptosis, and may be also related to differentiation of the radial glial cells. Therefore, we assessed hippocampal GFAP levels by immunoblotting (Figure 6). After KA-treatment, the level of GFAP was significantly increased at day 3, 5, and 7 post KA-injection. Full length GFAP (52 kDa) was progressively cleaved into smaller fragments time-dependently (Figure 6a). GFAP fragments of 45 kDa at day 1 post KA-injection and 40 kDa at day 3 post KA-injection were detected. The level of 45 kDa fragment increased from 4.61 ± 1.68% in the control to 82.06 ± 12.84%, 116.69 ± 30.81%, 124.58 ± 27.29% and 149.97 ± 36.75% of the control at day 1, 3, 5, and 7 post KA-injection, respectively. The level of 40 kDa fragment increased to 3.59 ± 1.68%, 31.63 ± 25.20%, 52.58 ± 21.65% and 87.28 ± 38.79% of 52 kDa GFAP in the control at day 1, 3, 5, and 7 post KA-injection, respectively. The co-injection of KA and caspase 3 inhibitor inhibit 40 kDa fragment production at day 7 post KA-caspase 3-injection (Figure 6b).

### Neuroplasticity is modulated in the hippocampus of KA-icv-injected mice

**Figure 7:**
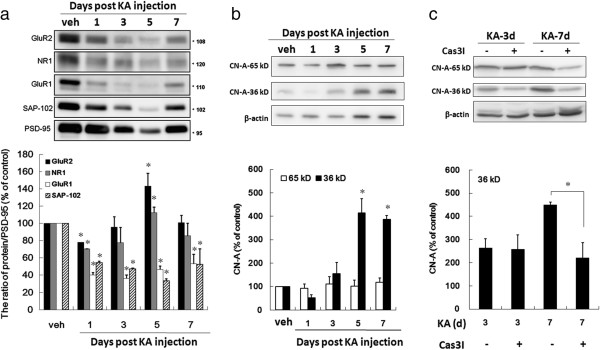
**The alteration of the neuroplasticity-related proteins in the hippocampus of KA-icv-injected mice is caspase 3 dependent.** CD-1 mice received KA-icv-injections and were sacrificed at day 1, 3, 5, and 7 post KA-injection. For control, the mice were sacrificed at day 7 post vehicle-injection (veh). Hippocampus The homogenate and PSD domain of hippocampus were prepared. The GluR2, GluR1, NR-1 and SAP-102 of PSD domain **(a)** and CN-A of total hippocampal homogenate **(b)** were analyzed by immunoblotting. **(c)** CD-1 mice received KA-icv-injections alone or co-injection of KA and caspase 3 inhibitor (Cas3I) or vehicle (veh), and were sacrificed at day 3 or 7 post KA-injection (KA-d3 and KA-d7). Hippocampal lysates were analyzed by immunoblotting. PSD-95 and β-actin were used as internal standard for **(a)** and **(b, c)**, respectively. The top part is the representative immunoblots. The bottom part in **(a)** is the ratio of proteins/PSD-95 relative to the ratio of the control (veh). The bottom part in **(b)** and **(c)** show the percentage of the level of 65 kD (open circles) and 36 kD (closed circles) relative to the level of 65 kD in the control (veh). Results are mean ± S.D. from five independent experiments. Significant differences between the control (veh) and KA injection in **(a, b)** are indicated by *, P < 0.001. Significant differences between the mice injected with and without caspase 3 inhibitor in **(c)** are indicated by *, P < 0.001.

Immunoblot analysis of PSD-proteins from the brain homogenates after KA-injection showed a time-dependent decrease of the level of PSD-95, NMDA receptor (NR)1, glutamate receptor (GluR)1, GluR2 and SAP-102 from day 1 to 5 post KA-injection, which are partially recovered at day 7 post KA-injection (Figure 7a). To compare the synaptic removal extent of different PSD proteins, the decrease of NR1, GluR1, GluR2 and SAP-102 are compared to that of PSD-95. The result shows that the ratio of NR1/PSD-95 and SAP-102/PSD-95 is significantly decreased during the period of day 1 to 7 post KA-injection. The ratio of GluR1/PSD-95 and GluR2/PSD-95 is significantly decreased and increased at day 1 and day 5 post KA-injection, respectively (Figure 7a).

Synaptic removal of α-amino-3-hydroxy-5-methyl-4-isoxazolepropionic acid receptors (AMPARs) has been reported to be mediated by calcineurin A (CN-A) which is proteolytically activated by caspase 3. Therefore, the KA injection-mediated proteolysis of CN-A were examined (Figure 7b). The result shows that the active form CN-A (36 kDa fragment) increased to 415.09 ± 59.33 and 387.37 ± 15.84% of the vehicle injection at day 5 and 7 post KA-injection, respectively. To verify the involvement of caspase 3 on KA-induced proteolysis of CN-A, caspase 3 inhibitor were co-injected with KA and the result shows that the level of active form CN-A (36 kDa fragment) at day 7 post KA-injection is significantly decreased (Figure 7c).

### Neurogenesis is significantly promoted in the hippocampus of KA-icv-injected mice

**Figure 8:**
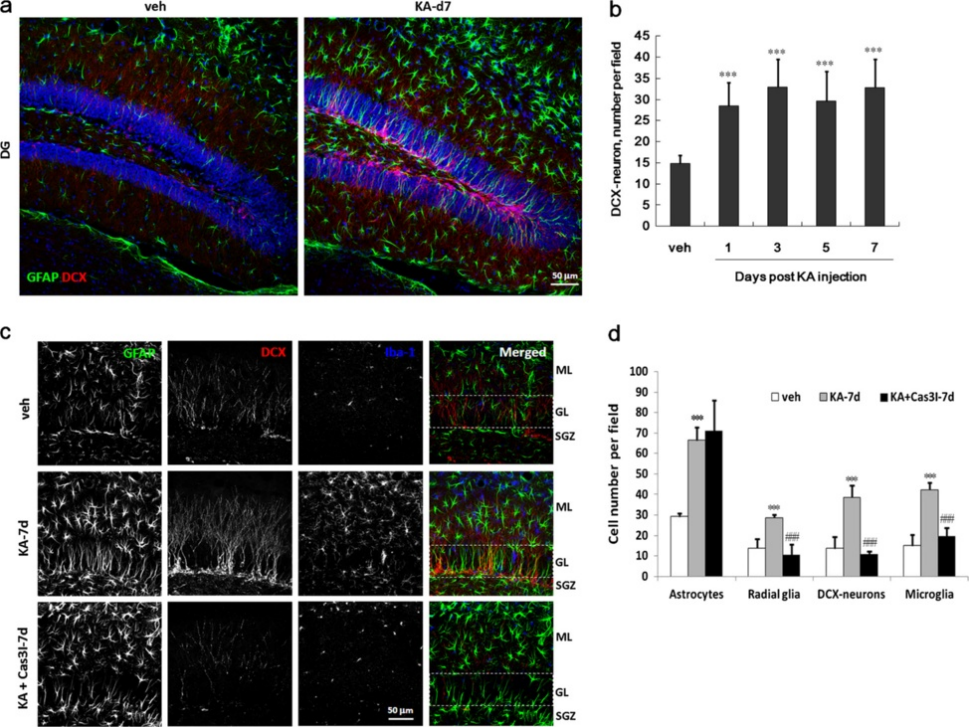
**Caspase 3 inhibitor prevents the KA-promoted hippocampal neurogenesis and microglial activation, but not astrogliosis, in the KA-icv-injected mice.** CD-1 mice received KA-icv-injections and were sacrificed at day 1 to 7 post KA-injection. For control, the mice were sacrificed at day 7 post vehicle-injection (veh). **(a and b)** The neurogenesis and astrogliosis in the ipsilateral side of DG was examined by anti-DCX (red) and anti-GFAP (green) antibodies. Cell nuclei were stained with Hoechst 33258 (blue). Panel **(a)** shows the representative GFAP and DCX fluorescent images of DG at day 7 post KA-injection as compared with vehicle injection. Panel **(b)** shows the calculated number of DCX-positive neurons in 250 μm × 250 μm field. The data are represented as the percentage related to the control (veh). **(c and d)** CD-1 mice received KA-icv-injections alone (KA-7d) or co-injection of KA with caspase 3 inhibitor (KA + Cas3I-7d) or vehicle (veh), and were sacrificed at day 7 post KA-injection. The neurogenesis, microglial activation and astrogliosis in the suprapyramidal blade of DG was examined by anti-DCX (red), anti-GFAP (green) and anti-Iba-1 (blue) antibodies, respectively. Panel **(c)** shows the representative fluorescent images of GFAP, DCX and Iba-1 of DG at day 7 post KA-injection as compared with vehicle injection. The dotted lines define the molecular layer (ML), granular layer (GL) and subgranular zone (SGZ) of DG. Panel **(d)** shows the calculated number of DCX-positive neurons in the 250 μm × 250 μm field. The results are the mean ± S.D. from 8 images. Significant differences between the control (veh) and the KA injection are indicated by ***, P < 0.001. Significant differences between the KA injection alone and the co-injection of KA and caspase 3 inhibitor are indicated by ###, P < 0.001.

We hypothesized that the temporal- and spatial-dependent decline of FJB-positive signal in DG may be attributable to neurogenesis in SGZ. Therefore, we assessed newly born granular neurons and radial glial cells in the SGZ by immunostaining using anti-doublecortin (DCX) and anti-GFAP antibody, respectively. The result shows that the number of DCX-positive newly born neurons and radial glial cells in SGZ are increased after KA injection (Figure 8a and 8b). Both newly born neurons and radial glial cells are increased in the period of day 1 to 7 post KA-injection (Figure 8b).

### Caspase inhibition prevents the promotion of neurogenesis and microglial activation, but not astrogliosis, in the hippocampus of KA-icv-injected mice

The above results indicate that KA injection promotes caspase 3 activation and enhances neurogenesis, microglial activation, proliferation of radial glial cells and astrogliosis in the hippocampus of mice. We thus examine the role of caspase 3 activation at day 7 post KA-injection due to that time point presented all four events of neurogenesis, microglial activation, proliferation of radial glial cells and astrogliosis. The suprapyramidal blade of granular layer of DG was triple-immunostained using anti-DCX, anti-Iba-1 and anti-GFAP antibodies to determine the newly born neurons, microglia, astrocytes and radial glial cells, respectively (Figure 8c and 8d). The result showed that the KA-injection-mediated cell number increase of radial glial cells, newly born neurons, and microglia are diminished by caspase 3 inhibitor. The KA-injection-mediated astrogliosis is however not affected by caspase 3 inhibitor.

## Discussion

Our results demonstrate several novel-finding on caspase 3-involved neurodegeneration, synaptic plasticity, reactive gliosis, and neurogenesis in KA-mediated excitotoxicity. KA induced an acute neurodegeneration in hippocampus detected by FJB-staining which occurred in CA1/3-pyramidal neurons and DG-hilar neurons (Figure 1a, b). The similar FJB-labeled neurodegeneration was also observed in the previous study [37]. Another type of delayed and irreversible neurodegeneration in CA3 and DG-hilus was also detected by the loss of NeuN expression (Figure 1a, c). KA induces different neurodegeneration among CA1, CA3 and DG-hilus regions which may be due to that the stratum lucidum region of CA3 is highly enriched with high-affinity KA binding sites [38]. CA3 neurons are directly excited by stimulation of their KA receptors and indirectly, by increased glutamate efflux secondary to KA stimulation of mossy fibers [39,40]. Therefore, the transient neurodegeneration detected by the loss of NeuN expression in CA1 may be also derived from the mild insult in CA1. It is puzzling that the neurodegeneration (NeuN-loss) occurs transiently in CA1 area at day 5 post KA injection. It can also be explained by KA-induced a transient loss of NeuN mRNA or protein.

It is unclear whether microglial activation initiates the disease progression or that merely response to neuronal death [39]. In our present study, microglia are activated in two phase after KA-injection (Figure 3). The first phase activation may be derived from KA-mediated excitotoxicity that may subsequently affect neurogenesis [41-43], synaptic stripping [44,45], and gliosis [46,47]. Moreover, reactive glial cells produced pro- and anti-inflammatory cytokines, chemokines, neurotrophic factors and other modulators to be involved in neuron-glia communication. Previous studies have indicated that the initial limbic seizures increase hippocampal neurogenesis [14-17]. In our study, KA-mediated an acute neurogenesis include the proliferation of radial glial cells and increase of newly born neurons (Figure 7a, b).

KA-induced non-apoptotic caspsae 3 activation is both cell type and region specific (Figure 2) which is similar to the previous report [48]. KA-induced caspsae 3 activation in CA1 is located in the cell body of pyramidal neurons. In CA3, however, it is located in both cell body of pyramidal neurons and the neurites in stratum radiatum. Alternatively, caspase 3 activation is located in interneurons in DG-hilus. The different onset of caspase 3 activation in CA1, CA3 and DG-hilus may due to that CA3 is highly accessible to KA-mediated excitotoxicity [38]. A similar nuclear activation of caspase 3 in neurons has been described previously [49,50]. Those studies suggested that SE-mediated nuclear caspase 3 activation may activate caspase-activated DNase (CAD) results in DNA fragmentation and apoptosis [51]. However, caspase 3 activity may be suppressed by inhibitors of apoptotic proteins (IAP), which promotes neuronal survival [52,53]. Previous study indicated that apoptotic caspase 3 activation following SE [32,33,48]. However, they found that caspase 3 contributes to the cell death in only a small proportion of degenerating cells. Henshall et. al. [32] claimed that caspase 3 may play a significant role in the mechanism by which neurons die following seizures. They also found that caspase-3 inhibitor significantly improved neuronal survival following seizures. However, in their IHC data, the TUNEL-neurons were colocalized with the IR of caspase 3, but not the activated caspase 3. Therefore, the increased caspase3-IR may not reflect the activated caspase 3 in the apoptotic neurons.

The non-apoptotic caspase 3 activation may also involve in astrocyte differentiation [54,55]. Cleaved GFAP has been previously detected during neurotoxic process, and GFAP fragments have also been identified as the head domain cleaved GFAP by calpain or caspase 3 [27,29]. We assessed hippocampal GFAP levels by immunoblotting (Figure 6a). GFAP-IR was significantly increased after KA-injection. Full length GFAP (52 kDa) was progressively cleaved into smaller fragments of 45 kDa and 40 kDa by calpain and caspase 3, respectively. The KA-mediated production of 40 kDa fragment was inhibited by the co-injection of caspase 3 inhibitor (Figure 6b) confirm the involvement of caspase 3.

The express of active caspase 3 in the GFAP-positive radial glial cells increased after KA-injection suggests that caspase 3 functions as a regulatory molecule in neurogenesis [56,57]. The co-injection of caspase 3 inhibitor prevent KA-mediated increase of radial glial cells, newly born neurons, and activated microglia, but not the astrogliosis, suggesting that astroglial caspase 3 was activated after gross astrogliosis, which then regulate microglial activation and neurogenesis. Microglia has been described to be a mediator of neurogenesis [42]. Thus the first phase microglial activation may involve the KA-mediated neurogenesis, and which was inhibited by caspase 3 inhibitor. Further investigation is needed to determine key components of this potential mechanism of astrogliosis, including the subcellular localization of active caspases, the identity of relevant transcription factors, and the relationships between these components and regulation of specific factors such as glutamine synthetase and fibroblast growth factor-2 [57]. In the reactive neonatal astrocyte cultures, specific inhibition of caspases 3 attenuated glutamine synthetase and fibroblast growth factor-2 expression, but did not reverse the morphological reactive phenotype may explain why caspase 3 inhibitor did not alter astrogliosis in our study.

Caspase 3 has been implicated in synaptic remodeling [24,28,58], cell differentiation [26,59], and cytoskeletal remodeling [25,60,61]. We detected the non-apoptotic caspase 3 activities in post synaptic density, which triggered the dominant removal of the GluR1 and SAP-120, but not GluR2 and NR1 from postsynaptic sites. These molecular modifications may correlate with spine degeneration [62]. Proteolytic cleavage of CN-A was significantly reduced when caspase 3 was inhibited, confirming the direct involvement of caspase 3 activation in the dendritic plasticity alteration and astrogliosis after KA-injection. These molecular modifications may correlate with spine degeneration which was observed via the GluR1 removal from PSD and the proteolytic activation of CN-A in hippocampus [63]. Our study is the first time to reveal a caspase 3-dependent mechanism that contributes to synaptic molecular change in KA-mediated neurotoxicity. A similar synaptic modification was described in a mouse model of Alzheimer’s disease [64].

The KA-mediated neuroplasticity alteration was detected by examining the loss of the PSD-proteins, especially the GluR1, in the PSD domain. Therefore, the activation of caspase 3 may be important for the integrity of neurites which is required for the neuroplasticity. However, the neuritic structures were disturbed seriously by the injection of KA combined with caspase 3 inhibitor but not by KA alone (Figure 5). Therefore, neuroplasticity alteration was detected by the proteolytic activation of CN-A. The result indicated that the proteolytic activation of CN-A was inhibited by the co-injected caspase 3 inhibitor (Figure 6b, c).

## Conclusions

Our results provide the first direct evidence of a causal role of caspase 3 activation in the cellular changes during KA-mediated excitotoxicity. These findings may highlight novel pharmacological strategies to arrest disease progression and control seizures that are refractory to classical anticonvulsant treatment.

## Competing interests

The authors declare that they have no competing interests.

## Authors’ contributions

TTT and HJT participated in the design of the study and performed the statistical analysis. LC, CLH and THL carried out the animal experiment and immunoassays. FLH participated in figure editing. YJS participated in its design and coordination and helped to draft the manuscript. All authors read and approved the final manuscript.
